# Reviewers BJCVS 32.1

**Published:** 2017

The Brazilian Journal of Cardiovascular Surgery (BJCVS) is grateful for the reviewers, listed below, which collaborated in this edition. Without their work would be impossible to keep the high scientific standard of our journal.

Bruno Rocha
Carla Tanamati
Edmo Gabriel
Edward Gologorsky
Guilherme Succi
Henrique Murad
João Carlos Ferreira Leal
José Glauco Lobo Filho
Magaly Arrais dos Santos
Mauro Paes Leme De Sá
Nelson Hossne
Paulo Roberto Evora
Rui M. S. Almeida
Sergio Francisco dos Santos Junior

